# Rare Presentation of Thyrotoxicosis Defying Methimazole Treatment: A Case Series

**DOI:** 10.7759/cureus.58980

**Published:** 2024-04-25

**Authors:** Mahvish Renzu, Vidhi Mehta, Adam Qazi, Alexander M Satei, Wael Taha

**Affiliations:** 1 Internal Medicine, Trinity Health Oakland, Pontiac, USA; 2 Internal Medicine, Trinity Health Mid-Atlantic, Darby, USA; 3 Internal Medicine, Detroit Medical Center/Wayne State University, Detroit, USA; 4 Radiology, Trinity Health Oakland, Pontiac, USA; 5 Endocrinology, Detroit Medical Center/Wayne State University, Detroit, USA

**Keywords:** thyrotoxicosis, management of thyrotoxicosis, graves’s diseases, methimazole resistance, antithyroid medication resistance, antithyroid medications, methimazole, treatment of hyperthyroidism, hyperthyroidism, endocrine disorders

## Abstract

Thyrotoxicosis, also known as hyperthyroidism, is a condition characterized by the excessive production of thyroid hormones by the thyroid gland. Besides Graves' disease, other common causes of thyrotoxicosis include toxic multinodular goiter, toxic adenoma, and subacute thyroiditis. The treatment of thyrotoxicosis depends on the underlying cause and may include medications (e.g., antithyroid drugs, beta-blockers), radioactive iodine therapy, or surgical removal of the thyroid gland (thyroidectomy). In this report, we present two instances of thyrotoxicosis where conventional high doses of antithyroid treatment failed to control the condition effectively. This failure prompted the exploration of alternative therapeutic interventions. These cases highlight the intricacies involved in managing thyrotoxic crises that do not respond to methimazole (MMI), emphasizing the necessity for innovative approaches such as plasmapheresis and thyroidectomy. Understanding such scenarios is vital for enhancing the care provided to patients encountering resistance to standard treatments. The distinct clinical pathways and treatment strategies adopted in these cases offer valuable insights into this disease management, particularly concerning resistance to MMI.

## Introduction

Thyrotoxicosis is a condition marked by excessive thyroid hormone activity, often stemming from elevated levels of thyroid hormones in the bloodstream. It affects approximately 2% of women and 0.2% of men [1]. Its clinical presentation can vary greatly, from mild symptoms to severe thyroid storms, which can be life-threatening. The primary method for managing thyrotoxicosis is by inhibiting thyroid hormone production using antithyroid medications, radioiodine ablation, or thyroidectomy [2]. The most used antithyroid medication is methimazole (MMI), a thioamide medication. It works by inhibiting thyroid peroxidase, a pivotal enzyme for synthesizing thyroid hormones. This mechanism decreases the synthesis of thyroid hormones, specifically thyroxine (T4) and triiodothyronine (T3), and it restores normal thyroid function [3]. While MMI is often effective, there are instances of resistance, posing a significant challenge in managing hyperthyroidism [4]. Some prior case reports of hyperthyroidism have shown resistance to standard doses of MMI, but the underlying mechanisms of this resistance remain unclear. While MMI remains the cornerstone of treatment, alternative therapeutic options (e.g., radioactive iodine therapy or thyroidectomy) may be necessary in cases of resistance. Early recognition and appropriate intervention are crucial to optimizing outcomes in patients with this challenging condition.

## Case presentation

Case 1: A 68-year-old white male, with a past medical history of hypertension and Parkinson's disease, presented with complaints of generalized weakness and left-sided chest discomfort. An electrocardiogram (ECG) revealed atrial fibrillation with a heart rate (HR) fluctuating between 180 and 200 beats per minute. He was initially treated with Cardizem and adenosine, which were ineffective. However, Lopressor helped bring his HR slightly down, and further lab workup revealed thyroid-stimulating hormone (TSH) < 0.01 mIU/L, free thyroxine (fT4) level was 5.9 ng/dL (reference range: 0.8-1.8 ng/dL), and the total triiodothyronine (tT3) level was 296 ng/dL (reference range: 80-200 ng/dL). The patient had no history of thyroid disorder, no recent use of contrast or iodine, and TSH receptor antibodies (TSHR Ab, also known as TRAb) were negative. However, he had chronic tremors from Parkinson's disease. He had also experienced weight loss without dieting in the past few weeks and denied experiencing diarrhea or constipation as well as any skin or hair changes. No sign of Graves’ orbitopathy. Other biochemical laboratory results were within the normal range.

**Figure 1:**
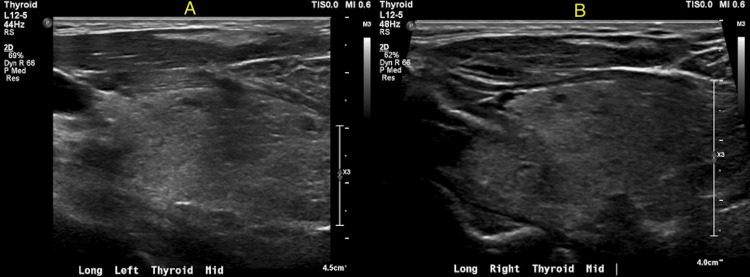
Greyscale images of the left (1A) and the right (1B) thyroid gland demonstrate diffusely heterogeneous echotexture of the thyroid gland without discrete nodularity.

The ultrasound (US) of the neck showed a heterogeneous thyroid gland suggestive of chronic thyroid disease, without any discrete thyroid nodule (Figures 1A-1B). Hence, the patient was diagnosed with thyrotoxicosis with a crisis. He was admitted to the ICU, where a beta-blocker was administered, followed by the initiation of MMI 30 mg daily. The patient persistently remained in tachycardia and complained of feeling warm. The MMI dose frequency was slowly up-titrated to 20 mg twice daily, followed by 20 mg thrice daily. On the third day of admission, the patient's symptoms began to improve, but he persistently had increased fT4 levels. Consequently, iodine therapy 250 mg four times daily was initiated, and alternative options such as thyroidectomy were discussed. Despite these interventions, the patient's fT4 levels continued to increase with no further improvement (Table 1). Hematology was consulted, and he underwent five sessions of plasmapheresis, after which his fT4 improved mildly. Given the resistance to MMI and iodine treatment, the patient underwent thyroidectomy, resulting in a decrease in fT4 levels to subnormal levels and an increase in TSH levels. The biopsy showed scattered histiocytes and giant cells, observed amidst follicles and luminal colloids within the gland, suggesting a nonspecific reaction pattern possibly linked to drug exposure or chronic medical conditions. The patient was started on levothyroxine and advised to follow up with labs in two to three weeks.

**Table 1:** Levels of free thyroxine (T4) over several days of admission along with the corresponding reference range.

Day of admission	T4 (thyroxine) free	Reference range (ng/dL)
Day 1	5.90	0.61-1.24
Day 3	>6	0.61-1.24
Day 6	5.60	0.61-1.24
Day 10	4.46	0.61-1.24
Day 15	0.77	0.61-1.24

Case 2: A 65-year-old African American woman, with a history of multiple lumbar disc herniations, had been bedridden for nine years, resulting in residual weakness in her right arm and a sacral pressure ulcer. She arrived at the hospital with altered mental status and was diagnosed with septic shock, characterized by an HR of 170 beats per minute and a temperature of 101.3°F. She was admitted to the ICU for further management and eventually required intubation.

**Figure 2:**
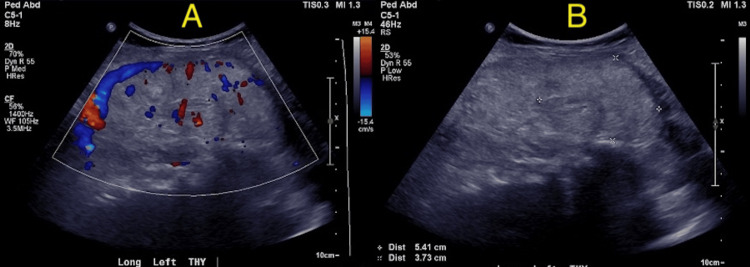
Color Doppler image of the left thyroid nodule in (1A) demonstrates internal vascularity of the left thyroid nodule, and image (1B) shows a solid, isoechoic, wider-than-tall lesion with smooth margins and no echogenic foci measuring 5.4 x 3.7 x 2.7 cm within the inferior pole of the thyroid gland. This lesion is classified as a thyroid imaging reporting and data system 3 (TI-RADS 3) lesion.

Further workup showed TSH < 0.01 mIU/L, fT4 2.16 ng/dL, tT3 67 ng/dL, and negative TRAb. There was no reported history of previous thyroid disease; however, significant weight loss recently associated with a change in mental status was reported. No signs of Graves’ orbitopathy were noted. The ultrasound of the neck showed bilateral isoechoic thyroid nodules, with the largest on the left measuring 5.4 cm (Figures 2A-2B). Because of concerns for thyroid storm, as indicated by a high thyroid storm score, IV steroids were administered, followed by daily MMI 20 mg. The patient's T4 levels were highly fluctuating but consistently high (Table 2). MMI was up-titrated to 20 mg thrice daily with propranolol. Despite high-dose therapy, the patient had persistently elevated T4 levels. Concerns for MMI resistance prompted an increase in the dose to 30 mg thrice daily, followed by 40 mg thrice daily. Despite these measures, MMI therapy failed, and the patient remained unable to be extubated. A decision was made for tracheotomy with thyroidectomy on day 20 of admission, with the addition of Lugol’s solution before thyroidectomy. Following thyroidectomy, MMI and Lugol’s solution were discontinued, and T4 levels were reduced to 0.69 ng/dL (see Figure 4). A biopsy confirmed it to be a benign multinodular goiter. Once the TSH level increased, levothyroxine 50 mcg was initiated, and the patient was advised to follow up with labs in two to three weeks.

**Table 2:** This table displays the levels of free thyroxine (T4) over several days of admission, along with the corresponding reference range.

Day of admission	T4 (thyroxine) free	Reference range (ng/dL)
Day 1	2.16	0.61-1.24
Day 2	2.21	0.61-1.24
Day 4	2.41	0.61-1.24
Day 6	2.06	0.61-1.24
Day 8	1.75	0.61-1.24
Day 12	0.69	0.61–1.24

After the thyroidectomy, both the patient's symptoms of thyrotoxicosis resolved, with normalization of thyroid hormone levels. Patients were advised to follow up with laboratory tests in two to three weeks to monitor thyroid function and adjust levothyroxine dosage if necessary. Long-term follow-up appointments were scheduled to ensure that their thyroid hormone levels remained within the normal range and to monitor for any potential complications related to thyroidectomy or thyroid hormone replacement therapy.

## Discussion

Drug resistance presents a widespread obstacle in combating infectious diseases, but its impact can extend to the management of noncommunicable diseases. Specifically, antithyroid drug (ATD)-resistant thyrotoxicosis, while rare, has garnered attention primarily through case reports, highlighting its rarity within the medical literature. Notably, substantial knowledge gaps persist concerning the mechanisms underlying antithyroid drug resistance (ATDR) as well as the development of accurate diagnostic approaches for identifying this resistance phenomenon. Additionally, the optimal strategies for managing ATD-resistant thyrotoxicosis remain elusive, further complicating clinical decision-making in such cases [5].

The principal means of managing thyrotoxicosis is the inhibition of excess thyroid hormone production using antithyroid drugs, radioiodine ablation, or thyroidectomy [6]. Antithyroid drugs can be broadly categorized into two types based on their chemical structure: thiouracil and imidazoles. Propylthiouracil (PTU) and 2-methyl mercaptoimidazole (MMI) are the conventionally available members of the former and latter types, respectively [7]. Among these two, MMI is often preferred initially because of its effectiveness, ease of use, and relatively low cost compared to other treatment options [8]. PTU is typically reserved as a second-line agent, except in certain circumstances, such as during the first trimester of pregnancy, when it is preferred because of its lower risk of crossing the placenta and causing fetal thyroid suppression. However, PTU use is limited in nonpregnant individuals because of reports of severe hepatotoxicity associated with its use [9].

Here we present two cases of thyrotoxicosis that demonstrated resistance to significantly higher doses of MMI, requiring dosing ranging from sixty to ninety mg a day, which normally are in the higher ranges. However, normally, it is dosed at 15 to 45 mg/day. There are also some reported cases of requiring up to 150 mg/day of MMI for symptomatic control; however, in our cases, we did not go over the ninety mg maximum because previous clinical cases have demonstrated a higher incidence of serious adverse events [10]. Situations such as these may occasionally arise when patients do not respond to recommended or even supra-therapeutic doses of MMI. This poses a challenge in the diagnosis of ATDR and ultimately managing thyrotoxicosis, which can be fatal if left untreated. The primary cause of thyrotoxicosis in patients with ATDR was identified as Graves' disease (GD) in 60% of the cases. However, it remains uncertain whether the association between ATDR and GD indicates a higher prevalence of GD compared to other causes of thyrotoxicosis or if there is simply an increased occurrence of GD itself, which is already the most common cause behind thyrotoxicosis [11].

The mechanism underlying ATDR is not fully understood, with several proposed etiologies. These include factors such as malabsorption, elevated metabolic rates, antibodies against the drugs, and abnormalities in intra-thyroid accumulation or action of the medications. Additionally, atypical iodine intake, prevalent in regions with diets rich in iodine-containing foods such as seaweed and seafood, can impact the absorption and metabolism of antithyroid medications such as MMI [12]. Assessment of resistant cases of GD should commence with evaluating patient adherence to medication. This can be achieved through direct questioning during initial and subsequent visits. Additionally, ruling out malabsorption is crucial, which can be done through careful history-taking and physical examination. The patients described in this report did not exhibit any symptoms or indications of concurrent gastrointestinal or liver disorders, which could potentially impact the pharmacokinetics of MMI. Following these steps, antidrug antibodies or drug levels can be measured to assess resistance. Another method involves conducting a perchlorate release test, observed clinically four hours after drug ingestion. However, access to pharmaceutical-grade perchlorate may be limited in some regions [13].

The treatment of ATDR GD is a subject of controversy, largely because of its rarity. The limited literature available reports varied approaches to management. These include transitioning from carbimazole (CBZ) to PTU or trying other medications such as steroids, lithium, or Lugol's iodine. Lithium carbonate and inorganic iodine are both used in the treatment of hyperthyroidism. Lithium carbonate inhibits the release of thyroid hormone from the thyroid gland, while inorganic iodine can reduce the synthesis of thyroid hormones [14]. Corticosteroids, on the other hand, primarily work by suppressing the conversion of the inactive thyroid hormone T4 to the active form T3 in peripheral tissues [15]. For nearly a century, Lugol's solution and other iodide-containing preparations have been utilized as adjunctive therapy in patients with thyroid crises scheduled for thyroidectomy. Iodide has been demonstrated to lower thyroid hormone levels and diminish blood flow within the thyroid gland. However, concerns about an "escape phenomenon" have arisen, as the iodide effect has been suggested to be only transient [16,17]. Plasmapheresis is another effective and quick procedure that involves separating blood from plasma outside of the body. Although data are limited, plasmapheresis has been shown to reduce serum total T4 30 times faster than conventional treatment for thyrotoxicosis, according to a few case series data [18]. Additionally, definitive therapy has been recommended with thyroid ablation or thyroidectomy. Moreover, in patients who warrant urgent thyroidectomy, a euthyroid state is ideal preceding the surgery to accomplish better results. Elective measures should be utilized as a bridge for thyroidectomy.

## Conclusions

This case underscores a significant but rare clinical scenario involving resistant thyrotoxicosis. Our patient displayed resistance to standard thyrostatic medications. We have also explored potential management strategies for such individuals. In complex cases such as this, definitive treatments such as radioactive iodine therapy or surgery become necessary. However, preparatory measures such as steroid therapy or Lugol's solution and plasmapheresis may be employed to optimize the outcomes of patients for these definitive interventions. These adjunctive treatments can help mitigate symptoms and stabilize thyroid function, preparing patients for more definitive forms of therapy.

## References

[1] Cooper DS (2003). Hyperthyroidism. Lancet.

[2] Burch HB, Cooper DS (2015). Management of Graves disease: a review. JAMA.

[3] Awosika AO, Singh G, Correa R (2023). Methimazole. StatPearls.

[4] Li H, Okuda J, Akamizu T, Mori T (1995). A hyperthyroid patient with Graves' disease who was strongly resistant to methimazole: investigation on possible mechanisms of the resistance. Endocr J.

[5] Bensenor IM, Olmos RD, Lotufo PA (2012). Hypothyroidism in the elderly: diagnosis and management. Clin Interv Aging.

[6] Papaleontiou M, Haymart MR (2012). Approach to and treatment of thyroid disorders in the elderly. Med Clin North Am.

[7] Cooper DS (2005). Antithyroid drugs. N Engl J Med.

[8] Pearce EN (2006). Diagnosis and management of thyrotoxicosis. BMJ.

[9] Wang MT, Lee WJ, Huang TY, Chu CL, Hsieh CH (2014). Antithyroid drug-related hepatotoxicity in hyperthyroidism patients: a population-based cohort study. Br J Clin Pharmacol.

[10] Ata F, Khan AA, Tahir S, Al Amer Z (2023). Carbimazole-resistant Grave's thyrotoxicosis is a diagnostic and therapeutic dilemma, case report with literature review. Int Med Case Rep J.

[11] Zava TT, Zava DT (2011). Assessment of Japanese iodine intake based on seaweed consumption in Japan: a literature-based analysis. Thyroid Res.

[12] Saleem T, Sheikh A, Masood Q (2011). Resistant thyrotoxicosis in a patient with graves disease: a case report. J Thyroid Res.

[13] Wolff J, Chaikoff IL, Goldberg C, Meier JR (1949). The temporary nature of the inhibitory action of excess iodide on organic iodine synthesis in the normal thyroid. Endocrinology.

[14] Calissendorff J, Falhammar H (2017). Lugol's solution and other iodide preparations: perspectives and research directions in Graves' disease. Endocrine.

[15] Chopra IJ, Williams DE, Orgiazzi J, Solomon DH (1975). Opposite effects of dexamethasone on serum concentrations of 3,3',5'-triiodothyronine (reverse T3) and 3,3'5-triiodothyronine (T3). J Clin Endocrinol Metab.

[16] Hope N, Kelly A (2016). Pre-operative Lugol's iodine treatment in the management of patients undergoing thyroidectomy for Graves' disease: a review of the literature. Eur Thyroid J.

[17] Xu T, Zheng X, Wei T (2023). Preoperative preparation for Graves’ disease. Front Endocrinol (Lausanne).

[18] Vinan-Vega M, Mantilla B, Jahan N, Peminda C, Nugent K, Lado-Abeal J, Rivas A (2020). Usefulness of plasmapheresis in patients with severe complicated thyrotoxicosis. Proc (Bayl Univ Med Cent).

